# microRNA-106b-mediated down-regulation of *C1orf24* expression induces apoptosis and suppresses invasion of thyroid cancer

**DOI:** 10.18632/oncotarget.4947

**Published:** 2015-07-22

**Authors:** Gianna Carvalheira, Bruno Heidi Nozima, Janete Maria Cerutti

**Affiliations:** ^1^ Genetic Bases of Thyroid Tumors Laboratory, Division of Genetics, Department of Morphology and Genetics, Universidade Federal de São Paulo, SP, Brazil

**Keywords:** C1orf24, NIBAN, FAM129A, miR-106b, follicular thyroid carcinoma and papillary thyroid carcinoma

## Abstract

We previously showed that *C1orf24* expression is increased in thyroid carcinomas. Nonetheless, the mechanism underlying *C1orf24* deregulation is not fully understood. It has been widely demonstrated that microRNAs are involved in post-transcriptional gene regulation in several diseases, including cancer. Using *in silico* prediction approach, five microRNAs that bind to the 3′-untranslated region (3′-UTR) of *C1orf24* were identified. The expression of two selected microRNAs (miR-17-5p, miR-106b) and the expression of *C1orf24* were tested in 48 benign and malignant thyroid lesions and in five thyroid carcinoma cell lines. miR-106b was down-regulated in thyroid cancer specimens and thyroid carcinoma cell lines, while *C1orf24* expression was markedly increased. To demonstrate that miR-106b reduces *C1orf24* expression, follicular (WRO) and papillary (TPC1) thyroid carcinoma cell lines were transiently transfected with miR-106b mimic. Ectopic expression of the miR-106b mimic significantly inhibits *C1orf24* mRNA and protein expression in both WRO and TPC1 cells. Dual-luciferase report assays demonstrated that miR-106b directly targets *C1orf24* by binding its 3′- UTR. Moreover, miR-106b-mediated down-regulation of *C1orf24* expression increased apoptosis and inhibited migration. We additionally demonstrated that siRNA against *C1orf24* significantly decreased its expression, inhibited cell migration and cell cycle progression while induced apoptosis. In summary, our findings not only provide new insights into molecular mechanism associated with *C1orf24* overexpression in thyroid carcinomas but also show that *C1orf24* might increase proliferation and cell migration. Thus, decreasing *C1orf24* levels, by restoring miR-106b function, may have therapeutic implications.

## INTRODUCTION

Thyroid nodules are common in clinical practice. With widespread use of high-resolution ultrasound, incidental thyroid nodules are being detected in up to 67% of adult population [1]. Once a thyroid nodule is detected, it is important to exclude malignancy. Although Fine-needle Aspiration (FNA) is the diagnostic standard in evaluating thyroid nodule, about 15 to 30% yield indeterminate cytology findings and patients are referred for diagnostic thyroid surgery [1–5]. As the majority of nodules prove to be benign on final histology, it is imperative to improve preoperative diagnostic evaluation.

In our previous studies, we show that the expression of three genes (*C1orf24*, *ITM1 and PVALB*) help to distinguish a benign from a malignant thyroid nodule with high sensitivity, specificity, positive predictive value and negative predictive value [6–8]. Among these thyroid markers, *C1orf24 (Chromosome 1 Open Reading Frame 24*), also called *FAM129A* and *NIBAN,* was found to be one of the best predictors of cancer. *C1orf24 was* highly expressed in follicular thyroid carcinoma (FTC) and papillary thyroid carcinoma (PTC), while was not expressed in normal thyroid and in benign follicular thyroid adenoma (FTA) and hyperplasia [6, 7, 9].

*C1orf24* was initially found as differentially expressed between two renal carcinoma cell lines, established from the same *Tsc2* knockout (Eker) rat model of hereditary renal carcinoma [10]. Later, it was demonstrated that *C1orf24* was expressed in early neoplastic lesion in Eker rats, while it was absent in normal rat kidney. Furthermore, *C1orf24* was reported to be expressed in other hereditary renal carcinoma models such as *Tsc2* and *Tsc1* knockout mice. Likewise, the expression of *C1orf24* was also found in sporadic human renal cell carcinomas, which have no loss of heterozigosity of the *TSC2* gene [11]. As *C1orf24* expression is commonly induced at early stages of renal carcinogenesis, independent of *TSC2* status, the authors proposed *C1orf24* as a new marker of renal carcinogenesis.

In 2006, the same group reported *C1orf24* expression in most thyroid carcinomas, including papillary microcarcinoma, and in a small subset of thyroid benign lesions with oxyphilic (Hürthe) cells [12]. This study not only corroborates our findings but, importantly, describes that *C1orf24* is upregulated from the early stages of papillary thyroid carcinogenesis and Hürthle tumors. Finally, *C1orf24* was found expressed in early stages of head and neck squamous cell carcinomas (HNSCC) and remained upregulated through the carcinogenesis progression while normal counterparts were negative [13].

Although *C1orf24* is described as highly expressed in several tumors subtypes, little is known about the molecular mechanism underlying its expression. Understanding how the expression of *C1orf24* is regulated is central to comprehend its role in cancer.

MicroRNAs (miRNAs) are small endogenous noncoding RNA molecules that have been acknowledged as post-transcriptional regulators of gene expression. By binding to the 3′-untranslated region (UTR) of their mRNA targets, miRNAs can induce mRNA degradation or blockade of mRNA translation [14–17]. miRNAs have important roles in regulating a wide range of cellular processes such as development, cellular differentiation, proliferation, apoptosis and metabolism. Indeed, aberrant expression of miRNAs has been associated with genesis and progression of a wide variety of cancers, including thyroid [18–21].

In the present study we sought to investigate whether miRNAs could be involved in the post-transcriptional regulation of *C1orf24* in thyroid cancer. We provide evidence that *C1orf24* expression is directly regulated by miR-106b. Further functional studies demonstrated that both miR-106b and siRNA knockdown of *C1orf24*, in two thyroid carcinomas cell lines, inhibited cell migration and cell cycle progression while induced apoptosis.

## RESULTS

### *In silico* prediction of putative miRNAs targeting *C1orf24*

**Figure 1:**
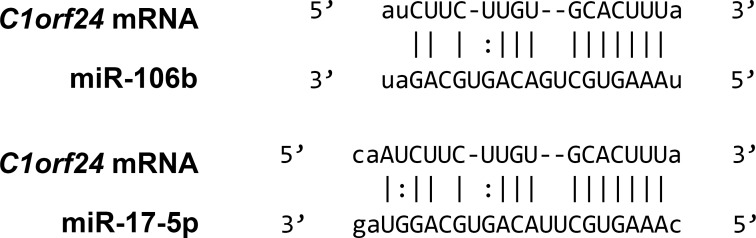
3′-UTR of *C1orf24* and predicted binding sites of miR-106b and miR-17-5p

Using *in silico* analysis, we identified five highly conserved miRNAs (miR-106b, miR-17-5p, miR-20a-5p, miR-106a-5p and miR-20b-5p) that potentially target 3′-UTR of *C1orf24* mRNA. miR-106b belongs to the miR-106b~25 cluster, which is located at 7q22.1. miR-17-5p and miR-20a-5p belong to the miR-17~92 cluster, which is located at 13q31.3. miR-106a-5p and miR-20b-5p are members of the miR-106a~363 cluster, which is located at Xq26.2. miRNAs were predicted by all three different target prediction programs (TargetScan, PicTar and miRanda). As miR-106a-5p and miR-20b-5p are located at X chromosome, and their variation could be due to sex-specific differential regulation of its expression, they were excluded from the analysis. Then, based on rank of prediction, one miR of each cluster was chosen for analysis. Therefore, miR-17-5p (*P* = −29.29 × 10^4^) and miR-106b (*P* = −25.10 × 10^4^) were chosen for further validation (Figure 1).

### Increased expression of *C1orf24* coincides with diminished expression of miR-106b in thyroid carcinomas

**Figure 2:**
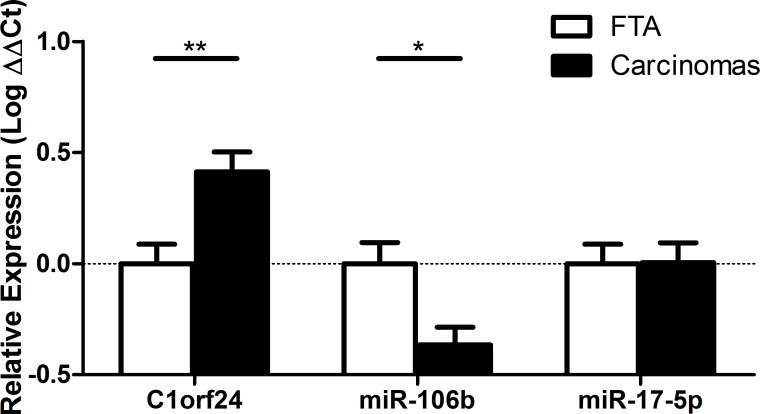
Expression of C1orf24 and selected miRNAs in follicular thyroid adenomas (FTA) and thyroid carcinomas *C1orf24* is overexpressed in thyroid carcinomas compared to FTAs (*P* = 0.0052). The expression of miR-106b is lower in thyroid carcinomas compared to FTAs (*P =* 0.0137). No difference was observed in miR-17-5p between FTAs and carcinomas. Data are presented as means ± SD (*n* = 48). *P* values represent statistical analysis using Mann-Whitney U test.

We previously reported that *C1orf24* was overexpressed in thyroid carcinomas at both mRNA and protein levels [6, 8]. The expression of *C1orf24* and selected miRNAs (miR-17-5p and miR-106b) were investigated by quantitative PCR (qPCR) in 48 thyroid samples. The expression of *C1orf24* was increased in nearly all thyroid carcinomas, while its expression was absent in most benign FTAs (*P* = 0.0052). In contrast, the expression of miR-106b was observed as consistently down-regulated in most thyroid carcinomas compared to benign FTAs (*P* = 0.0137) (Figure 2, Supplementary Figure S1). These data suggest that an increased expression of *C1orf24* upon malignant transformation coincides with a decreased expression of miR-106b, which may advocate an underlying association between these phenomena. The expression of miR-17-5p did not differ between FTAs and carcinomas.

### miR-106b is expressed at low levels in thyroid cancer cell lines while *C1orf24* is expressed at high levels

**Figure 3:**
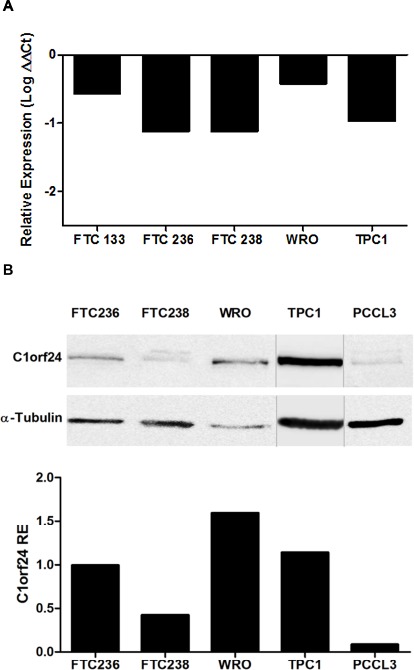
Endogenous expression of miR-106b and C1orf24 in thyroid carcinoma cell lines **A.** Quantitative PCR analysis showed that miR-106b expression is very low in all human thyroid carcinoma cell lines. ΔΔCt was calculated using FTA samples as the control group. **B.** C1orf24 expression in thyroid carcinoma cell lines and in a rat normal thyroid cell (PCCL3) by Western blot. α-tubulin was used as a loading control. The band intensities were quantified and normalized to α-tubulin intensities and the results are graphically represented (bottom).

We next determined the level of miR-106b expression in four follicular thyroid carcinoma cell lines (FTC 133, FTC 236, FTC 238, WRO) and one papillary thyroid carcinoma cell line (TPC1). qPCR analysis showed that miR-106b is expressed at very low levels in all cell lines (Figure 3A). We next investigated the C1orf24 expression in all thyroid carcinoma cell lines and in a rat normal thyroid cell line (PCCL3) at protein levels. *C1orf24* is expressed at higher levels in WRO and TPC1 cell lines (Figure 3B). These data are consistent with that observed in thyroid carcinoma specimens, where the miR-106b data are inversely correlated to the expression of *C1orf24*. Considering all thyroid carcinoma cell lines had comparable miR-106b expression (Figure 3A) and that WRO and TPC1 cell lines showed the highest expression levels of *C1orf24* (Figure 3B), these cell lines were chosen for further *in vitro* analysis.

### Ectopic expression of miR-106b in thyroid carcinoma cell lines inhibits *C1orf24* expression

**Figure 4:**
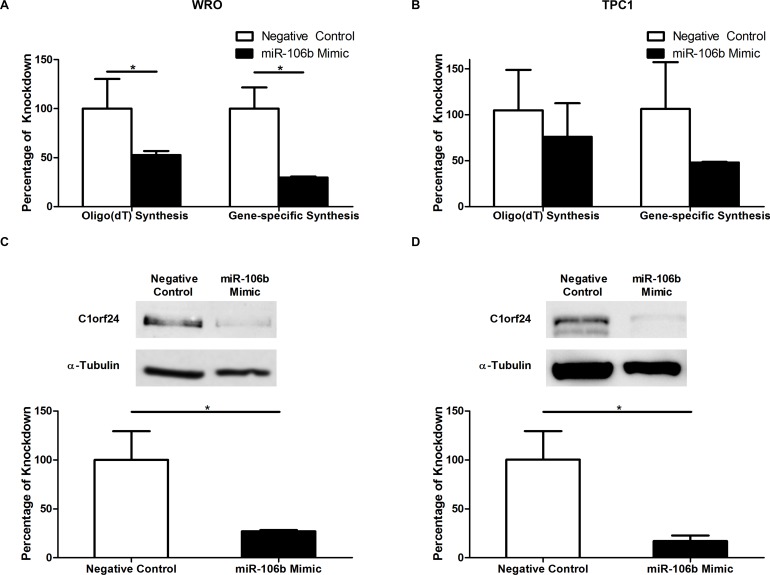
The effect of miR-106b on the expression of *C1orf24* Thyroid carcinoma cell lines were transfected with miR-106b and negative control mimics. *C1orf24* mRNA expression was measured in WRO (**A)** and TPC1 (**B)** cell lines by quantitative PCR using as template cDNA synthetized using oligo(dT)_12-18_ or *C1orf24* specific primers. The expression of *C1orf24* was lower after transfection with miR-106b mimic in both oligo(dT)_12-18_ (*P =* 0.0133) or primer specific (*P* = 0.028) analysis in WRO (**A)** and TPC1 (**B)** cell lines. C1orf24 protein levels were reduced in WRO (**C)** and TPC1 (**D)** transfected with miR-106b mimic (*P =* 0.0265 and *P =* 0.0486, respectively). The band intensities were quantified and normalized to α-tubulin and the results are graphically represented (bottom). Data are presented as means ± SD (*n* = 3), Mann-Whitney U Test.

To test if the miR-106b suppresses *C1orf24* expression, a small double-stranded RNA that mimics endogenous mature miR-106b was transfected into WRO and TPC1 cell lines. Cells were also transfected with a negative control, a random sequence which is not predicted to target any known gene. When the miR-106b expression was restored, abundance of C1orf24, at both transcript and protein levels, was reduced in WRO and TPC1 cell lines (Figure 4).

### *C1orf24* is a direct target of miR-106b

**Figure 5:**
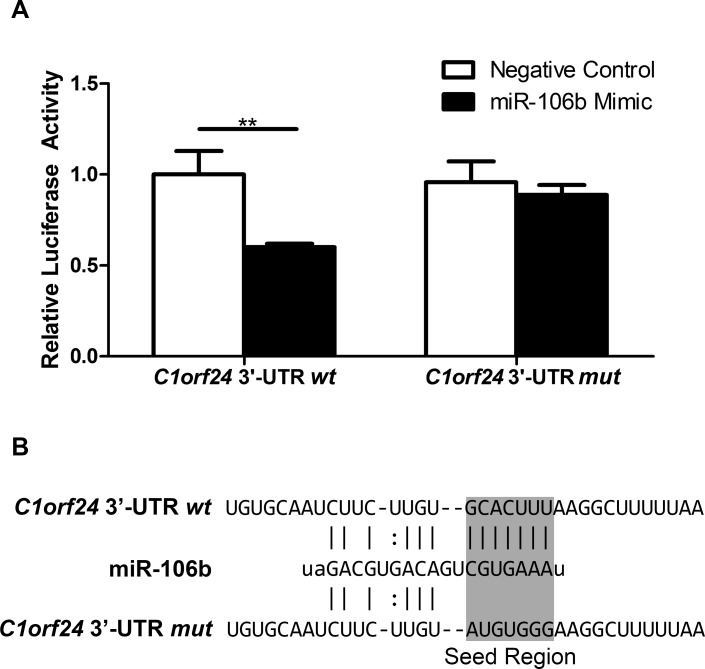
miR-106b negatively regulates *C1orf24* in thyroid cell line **A.** PCCL3 cells were co-transfected with pmirGLO *wt* or pmirGLO *mut* and miR-106b mimic or negative control. Luciferase activity was lower when vector carrying C1orf24 3′-UTR *wt* was co-transfected with miR-106b mimic, compared to the co-transfection of pmirGLO *wt* and negative control. When PCCL3 cells were co-transfected with vector carrying C1orf24 3′-UTR *mut* and miR-106b mimic or negative control, no differences were observed. Values are expressed as means ± SD (*n* = 6). **B.** 3′-UTR *C1orf24* mRNA showing wild type (*wt*) and mutated (*mut*) seed sequence which correspond to the binding site of miR-106b. **P < 0.01.

As ectopic expression of miR-106b led to suppression of *C1orf24*, luciferase assay was performed to investigate *in vitro* interaction between the miR-106b and *C1orf24* 3′-UTR. As endogenous expression of C1orf24 is very low in a normal rat thyroid cell line (PCCL3), this cell line was chosen (Figure 3B). The miR-106b binding site in the *C1orf24* 3′-UTR was cloned downstream of Firefly Luciferase gene. Co-transfection of the miR-106b mimic with reporter construct led to a drastic reduction of the luciferase activity, compared to the co-transfection of miR-106b mimic and negative control (*P* = 0.004, Figure 5A, left graphic). To confirm the interaction of miR-106b and *C1orf24* 3′-UTR, a plasmid carrying a mutation in the putative miR-106b binding site was used (Figure 5B). When the reporter construct carrying *C1orf24* 3′-UTR lacking the miR106 site (mutated) was co-transfected with the miR-106b mimic, the luciferase activity was similar to that observed in the cells co-transfected with the reporter construct with negative control (Figure 5A, right graphic). These results support the hypothesis that miR-106b directly interacts to and negatively regulates *C1orf24* expression by binding to its 3′-UTR mRNA.

### *C1orf24* knockdown, through ectopic expression of miR-106b, increases apoptosis rate and inhibits cell migration

**Figure 6:**
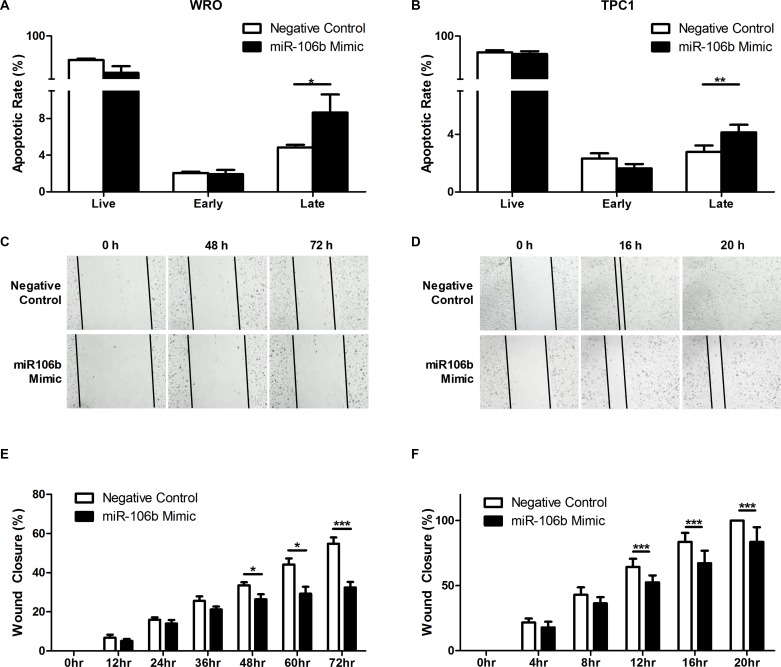
Effect of miR-106b in apoptosis and migration WRO (**A)** and TPC1 (**B)** cells transfected with miR-106b mimic increased late apoptosis rate compared to negative control (*P =* 0.0286 and *P* = 0.0082, respectively). Values are expressed as means ± SD (*n* = 5). Scratch wound assay was performed on WRO (**C** and TPC1 **D)** cells transfected with miR-106b or negative control. Data were collected over the indicated period of time and are graphically represented (**E** and **F)**. Values are expressed as means ± SD (*n* = 5). *P < 0.05 and *** P < 0.001, Student’s *T*-Test.

We here investigated whether ectopic expression of miR-106b, in WRO and TPC1 cell lines, induces apoptosis. The restoring of miR-106b expression in WRO and TPC1 cells increased apoptosis, compared to negative control (*P* = 0.0286 and *P =* 0.0082, respectively, Figures 6A and 6B). We further characterized the effect of *C1orf24* knockdown, through ectopic expression of miR-106b, on cell migration using wound-healing assay. After transient transfection, the extent of wound closure was monitored for up to 72 h in WRO and up to 48 h in TPC1. Representative images of two independent experiments performed in quintuplicate are shown in Figures 6C and 6D. Wound-healing assay showed that WRO and TPC1, transfected with miR-106b mimic, diminished scratch wounds closure compared to negative control (*P* = 0.0008 and *P =* 0.0119, respectively). Results are graphically represented in Figures 6E and 6F.

### siRNA-mediated knockdown of *C1orf24* increases apoptosis while inhibits cell migration and cell cycle progression

**Figure 7:**
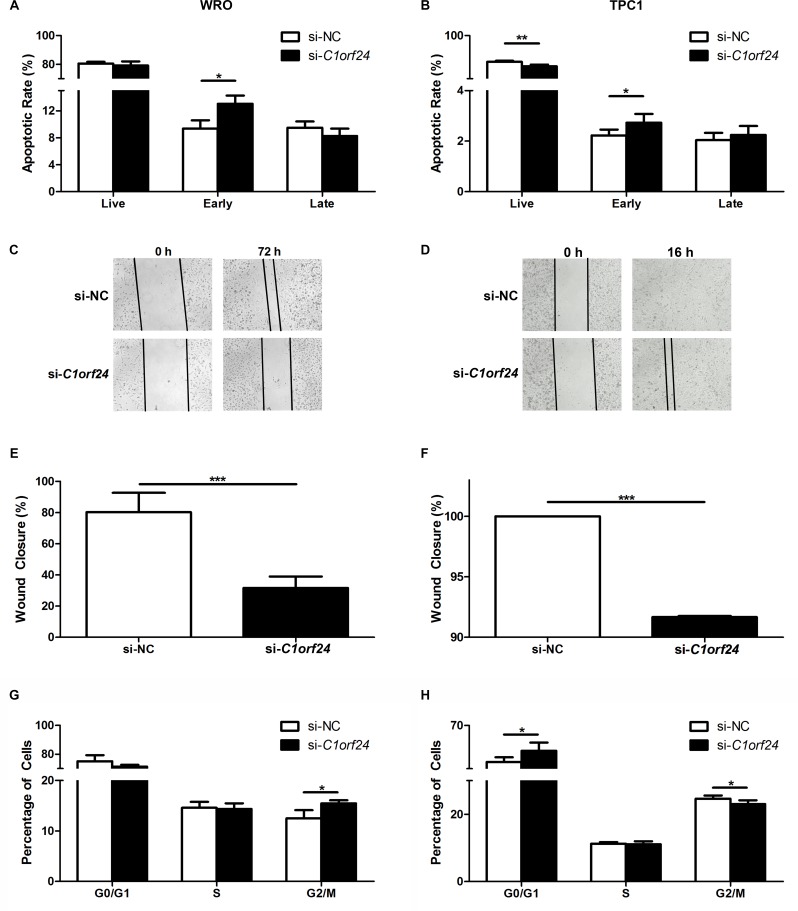
Effect of siRNA-mediated *C1orf24* knockdown in apoptosis, migration and cell cycle WRO (**A)** and TPC1 (**B)** cells transfected with siRNA specific to *C1orf24* (si-*C1orf24*) increased early apoptosis rate compared to negative control (si-NC, *P =* 0.0052 and *P* = 0.0317, respectively). Significant reduction in percentage of live TPC1 cells were detected after transfection with si-*C1orf24* (*P =* 0.0079). Values are expressed as means ± SD (*n* = 5). Scratch wound assay was performed on WRO (**C)** and TPC1 (**D)** cells transfected with si-*C1orf24* or si-NC. Data were collected over the indicated period of time and are graphically represented (**E** and **F)**. WRO **G.** cells transfected with si-*C1orf24* increased G2/M phase arrest compared to control (*P* = 0.033). G0/G1 phase arrest was observed in TPC1 cells (*P* = 0.029) (**H)**. Values are expressed as means ± SD (*n* = 5). ***P < 0.001, Student’s *T*-Test.

In order to check if the effects observed after miR-106b transfection into thyroid cell lines were due to *C1orf24* inhibition or due to an interaction with other gene, WRO and TPC1 cells were transiently transfected with small interfering RNA specific to *C1orf24* (si-*C1orf24*) or scrambled siRNA negative control (si-NC). Two siRNAs (si-*C1orf24*#1 and si-*C1orf24*#2*)* were able to knockdown the expression of C1orf24 (Supplementary Figure S2A). However si-*C1orf24*#1 showed greater knockdown efficiency and, therefore, was selected for further *in vitro* analysis (Supplementary Figure S2B). siRNA-mediated knockdown of *C1orf24* increased apoptosis in WRO (*P =* 0.0052) and TPC1 cells (*P =* 0.0317) (Figures 7A and 7B). We further characterized the effect of the si-*C1orf24* knockdown on cell migration using wound-healing assay. The extent of wound closure was monitored for up to 72 h in WRO and up to 48 h in TPC1. Representative images of two independent experiments are shown in Figures 7C and 7D. *C1orf24* knockdown inhibited scratch wounds closure at 72 h in WRO and at 16 h in TPC1 (*P* < 0.0001) (Figures 7E and 7F). To determine whether cell cycle was affected as a consequence of si-*C1orf24* knockdown, cell cycle progression was monitored. si-*C1orf24* knockdown increase the percentage of cells in the G2/M phase in WRO cell line (*P* = 0.033) and, the percentage of cells in G0/G1 in TPC1 cell line (*P* = 0.029) (Figures 7G and 7H).

## DISCUSSION

Our group previously identified *C1orf24* as one of the molecular markers differentially expressed between benign and malignant thyroid nodules and, therefore, a potential preoperative diagnosis marker [6–9]. Several studies showed that *C1orf24* is highly expressed in thyroid carcinomas [12] and other human cancers [10, 11, 13, 22]. However, the molecular mechanisms underlying its expression remain largely unknown.

It is known that miRNAs regulate gene expression at post-transcriptional level. Several studies have demonstrated that miRNAs repress the expression of oncogenes or tumor suppressor genes and, therefore, miRNA deregulation can contribute directly to the development of cancer, including thyroid cancer [21, 23–27]. Hence, we here investigated if miRNAs could down-regulate the expression of *C1orf24*.

Using an *in silico* analysis, five miRNAs, belonging to the miR-17 family, were predicted to target the *C1orf24* 3′-UTR site. The expression of the miRNAs miR17-5p and miR-106b were validated in thyroid samples. The expression of the miR17-5p did not differ between benign and malign tumors and, therefore, was not further explored. However, the expression of the miR-106b was significantly lower in thyroid carcinomas specimens and thyroid carcinoma cell lines than its expression in benign thyroid lesions. Moreover, an inverse correlation between *C1orf24* and miR-106b expression was observed in thyroid carcinomas, suggesting that miR-106b may attenuate *C1orf24* expression. Of note, miR-106b was recently described as one of 14 miRNAs that comprise the signature for classification of FTA and FTC [27].

We also showed that restoration of miR-106b expression, in WRO and TPC1 thyroid carcinoma cell lines, inhibited C1orf24 expression at both mRNA and protein levels. As our findings suggest that miR-106b mimic can promote *C1orf24* mRNA degradation, we used *C1orf24*-specific oligonucleotide to prime first-strand cDNA and to evaluate the *C1orf24* expression by qPCR. The miR-106b mimic represses *C1orf24* expression at similar rate to that observed when cDNA was primed using oligo-dT oligonucleotide (Figures 4A and 4B). These findings indicate that miR-106b can decrease C1orf24 expression by either inhibiting translation or directly causing degradation of its mRNA.

Additionally, we employed luciferase reporter-gene assay to confirm *C1orf24* as a target gene of miR-106b. Reduced luciferase activity confirmed that miR-106b binds to *C1orf24* 3′-UTR cloned region. On the other hand, site-direct mutagenesis in the miR-106b-binding site did not change luciferase activity, demonstrating that mutations in this binding site abolished its regulation. Therefore, here provide evidences that miR-106b directly regulates *C1orf24*, validating the *in silico* prediction approach [27].

Although the role of *C1orf24* in cancer remains unknown, it has been shown that *C1orf24* knockdown, by RNA interference, induces apoptosis [28]. Moreover, ectopic expression of miR-106b in breast cancer cell lines represses tumor migration, invasion and proliferation [29]. Hence, we investigated the effects of *C1orf24* knockdown through both ectopic expression of miR-106b and siRNA on apoptosis and cell migration. Our *in vitro* data support the hypothesis that *C1orf24* inhibits apoptosis. Inhibition of *C1orf24* expression by restoring miR-106b expression in follicular and papillary thyroid carcinoma cell lines significantly promoted late apoptosis while siRNA knockdown induced early apoptosis. Differences in apoptotic response can be partially explained by silencing efficacy (78%, Figures 4C, 4D, and 95%, Supplementary Figure S2), once siRNA is a specific silencing sequence while miRNAs can target different genes. Additionally, we also demonstrate, for the first time, that the inhibition of *C1orf24* expression, via siRNA knockdown or miR-106b expression, reduced cell migration.

Lastly, *C1orf24* knockdown induced cell cycle arrest. Since miR-106b can target different genes known to be involved in cell cycle, we did not evaluate its effect on cell cycle regulation. Intriguingly, in the WRO cells, with mutant *P53*, the cell cycle arrest occurred at G2/M phase while in the TPC1 cells, with wild-type *P53* status, the cell cycle arrest occurred at G0/G1 phase. It is well know that the p53-independent apoptosis slow the process occurring after a G0/G1 phase arrest while the p53-dependent apoptosis is much more faster and is characterized by early and late apoptotic phases, taking place in cells arrested at G0/G1 phase [30].

The apparent association between *C1orf24*, *P53* and apoptosis has been previously suggested by Ji et al 2012 [31]. They have been described that AKT phosphorylates C1orf24 at S602 under stress conditions. Phosphorylated C1orf24 binds to NPM, thereby preventing NPM from binding to MDM2. The free MDM2 interacts with P53 promoting its degradation and, therefore, increasing cell survival. Accordingly, C1orf24 depletion induces p53 stabilization and increases cell apoptosis [31].

To the best of our knowledge, this study is the first to provide insights about regulation of *C1orf24* expression in cancer. The decreased miR-106b expression and increase *C1orf24* expression may have a synergistic effect during the development and progression of thyroid cancer. Accordingly, we believe that *C1orf24* could act as an oncogene while miR-106b functions as a tumor suppressor, although further analyses are needed. These findings may have important therapeutic implications, as *C1orf24* down-regulation would have anti-proliferative, anti-invasive and pro-apoptotic effects.

## MATERIALS AND METHODS

### Thyroid samples

A total of 48 thyroid samples were obtained from patients who underwent thyroid surgery between 2000 and 2010 at Hospital São Paulo (Universidade Federal de São Paulo) and Hospital das Clínicas (Universidade de São Paulo). Tissue specimens were frozen in liquid nitrogen immediately after surgical resection and stored at −80°C until use. Final histological classification was obtained from paraffin-embedded sections. The study included 12 follicular thyroid adenomas (FTA), 10 follicular thyroid carcinomas (FTC), eight classical papillary thyroid carcinomas (CVPTC) and 18 follicular variant of papillary thyroid carcinomas (FVPTC). For all samples, informed consent, approved by the Independent Ethical Committees of Universidade Federal de São Paulo and Universidade de São Paulo, was obtained.

### Cell lines

WRO (follicular thyroid carcinoma) cell line was maintained in Dulbecco’s modified essential medium (DMEM) supplemented with 10% fetal bovine serum (FBS) (Life Technologies, Grand Island, NY). FTC 133 (follicular thyroid carcinoma) was maintained in DMEM and Hamŕs F12 (1:1 mixture) supplemented with 10% FBS (Life Technologies). FTC 236 (follicular thyroid carcinoma) was maintained in DMEM and Hamŕs F12 medium (1:1 mixture) supplemented with 10% FBS (Life Technologies), thyroid stimulating hormone (TSH 1 mU/mL) and insulin (10 μg/mL) (Sigma-Aldrich, St. Louis, MO). FTC 238 (follicular thyroid carcinoma) was maintained in DMEM and Hamŕs F12 (1:1 mixture) supplemented with 5% FBS (Life Technologies). TPC1 (papillary thyroid carcinoma) was maintained in RPMI supplemented with 10% FBS (Life Technologies). PCCL3 cell line (normal rat thyroid) was maintained in Ham’s F12 medium (Life Technologies) supplemented with 5% FBS, TSH (1 mU/mL), hydrocortisone (10 ng/mL), transferrin (5 μg/mL) and insulin (10 μg/mL) (Sigma-Aldrich). All cell lines were maintained in a 5% CO_2_ and 37°C humidified incubator.

### miRNA selection

*In silico* prediction of miRNAs targeting *C1orf24* was performed using three miRNA databases and softwares: TargetScan (http://www.targetscan.org/vert_40/), PicTar (http://pictar.mdc-berlin.de/) and miRanda (http://www.microrna.org/microrna/home.do).

### RNA isolation and quantitative RT-PCR

Expression level of *C1orf24* was quantified by quantitative RT-PCR (qPCR) in all thyroid samples and thyroid carcinoma cell lines. Total RNA was isolated from thyroid samples and cell lines using TRIzol Reagents, according to manufacturer’s instructions (Invitrogen Corp., Carlsbad, CA). One microgram of total RNA from each sample was reverse transcribed into cDNA using oligo(dT)_12-18_ primers, as described [8]. cDNA was then diluted five-fold, and 1.0 μL aliquot of cDNA was used in a 12 μL PCR reactions containing SYBR Green PCR Master Mix (Applied Biosystems, Foster City, CA) and 10 μmol/L of each primer for target gene or reference gene (*RPS8*). PCR primers were as follow: *C1orf24* sense (5′-CCAGAACTTCCAGACCACCAA-3′) and antisense (5′-CGGAATGCAGCGGAAGATT-3′) and *RPS8* sense (5′-AACAAGAAATACCGTGCCC-3′) and antisense (5′-GTACGAACCAGCTCGTTATTAG-3′). To investigate the expression levels of miR-17-5p and miR-106b in thyroid samples and thyroid carcinoma cell lines, 100 ng of total RNA was reversed transcribed into cDNA using TaqMan MicroRNA Reverse Transcription Kit for hsa-miR-17-5p (assay ID 002308), hsa-miR-106b-5p (assay ID 000442) and RNU24 (assay ID 001001) (Applied Biosystems). qPCR was performed using an aliquot of cDNA and TaqMan Universal PCR master mix, according to manufacturer’s recommendation (Applied Biosystems). qPCR reactions were performed in triplicate and threshold cycle (Ct) was averaged (SD ≤1). In thyroid samples, fold changes were calculated according to the comparative ΔΔCt method, as described [6]. FTAs were used as control group.

### Western blot analysis

Western blot analysis was performed to assess *C1orf24* expression in thyroid carcinoma cell lines, as previously described [32]. Briefly, protein was isolated using an ice-cold buffer containing 50 mM Tris-HCl (pH 7.4), 100 mmol/L NaCl, 50 mM NaF, 1 mM NaVO4, 0.5% NP-40 and protease inhibitor cocktail (Roche, Mannheim, Germany). Lysate was centrifuged at 10.000 × g at 4°C, supernatant was collected and protein concentration was determined using Bradford assay (Sigma-Aldrich). About 50 μg of protein was loaded into a pre-cast NuPAGE 4-12% Bis-Tris Gel (Invitrogen Corp.) and transferred onto a polyvinylidene difluoride membrane (Amersham Biosciences, Piscataway, NJ). Membrane was then incubated with primary antibody overnight at 4°C. Antibodies against C1orf24 (1:200) [7] or anti-α-Tubulin (1:10.000; Sigma Aldrich) were used. Subsequently, membrane was washed and incubated with peroxidase-conjugated secondary antibodies at 1:10.000 dilution (DAKO, Glostrup, Denmark). Immune complexes were detected using SuperSignal West Pico Chemiluminescent Substrate (Pierce, Rockford, IL) and ImageQuant LAS 4000 imaging system (GE Healthcare, Waukesha, WI). The chemiluminescent signal was quantified with ImageQuant software (GE Healthcare).

### Ectopic expression of miR-106b mimic in thyroid carcinoma cell lines

To investigate the putative effect of miR-106b on *C1orf24* expression, WRO and TPC1 cell lines were transiently transfected the miR-106b double-stranded RNAs, which mimics pre-miR-106b precursor (assay ID PM 10067). The scramble control miRNA duplex (assay ID AM 17110) was used as negative control (Ambion, Foster City, CA, USA). WRO cell line (8 × 10^4^) was transfected with 30 nM of miR-106b or negative control mimics using siPORT neoFX transfection reagents, according to manufacturer’s instruction (Ambion). TPC1 cell line (2.5 × 10^5^) was transfected with 100 nM of miR-106b or negative control mimics by cell electroporation (150 V, 900 μF).

### Transient transfection of siRNA targeting *C1orf24* in thyroid carcinoma cell lines

To investigate the putative effect of *C1orf24* silencing, we transiently transfected two pre-designed validated small interfering RNAs (siRNA) for human *C1orf24* (si-*C1orf24*#1 and si-*C1orf24*#2) into WRO. These siRNAs and the scramble siRNA negative control were commercially obtained from Ambion. WRO and TPC1 cell lines were transiently transfected as described above.

### *C1orf24* knockdown analysis

To monitor the mRNA expression of *C1orf24*, cells were harvested 48 h (TPC1) and 72 h (WRO) after either transfection with miR-106b mimic, si-*C1orf24* or respective scramble negative controls. Total RNA was extracted using TRIzol Reagents (Invitrogen Corp.). First-strand cDNA was primed using oligo(dT)_12-18_, as described above. Subsequently, *C1orf24* expression was measured by qPCR and relative expression calculate as above [6]. To confirm down-regulation of *C1orf24* at mRNA levels, cDNA synthesis was performed using gene-specific primer cocktails, as described by Liles *et al* [33]. Primers for target (*C1orf24*) and internal control (*RPS8)* were used. Oligonucleotides were as follow: *C1orf24* antisense (5′-CGGAATGCAGCGGAAGATT-3′) and *RPS8* antisense (5-’GTACGAACCAGCTCGTTATTAG-3′). Threshold cycle (Ct) was averaged (SD ≤1). The knockdown ratio (% KD) was calculated using the 100.(2^ΔΔCt^) method, accordingly to manufacturers’ instructions (Ambion). Negative control was used as reference sample. Each analysis was performed in three experimental replicates with three technical replicates within each experiment. To monitor the expression of C1orf24 at protein level, cells were harvested 72 h after transfection with miR-106b mimic, si-*C1orf24* or respective scramble negative controls. Western blot analysis was performed as afore mentioned. Three technical replicates were performed and SEM±SD were used to statistical analysis.

### Generation of the construct containing a 3′-UTR region of the human *C1orf24*

To demonstrate a direct interaction between miR-106b and *C1orf24*, a 688-bp region of *C1orf24* 3′-UTR containing the potential miR-106b binding site, as predicted by the informatics analysis, was PCR-amplified using a cDNA from a follicular thyroid carcinoma. Primers were as follow: sense: 5′-CCGTCTAGAACGTGAAGGAGGGAGAAGGT-3′ and antisense: 5′-CCGTCTAGAGGATGAGTAA CAGGCCCAGA-3′ (with *Xba*I linkers underlined). Primers were designed using Primer3 program (http://www.ncbi.nlm.nih.gov/tools/primer-blast/). Then PCR product was cloned into pCR2.1-TOPO vector (Invitrogen Corp.), according to manufacturer’s instructions. To confirm the identity of PCR products, DNA isolated from pCR2.1-TOPO containing 3′-UTR of *C1orf24* was sequenced using ABI Prism BigDye Terminator Cycle Sequencing Kit (Applied Biosystems) as described [34]. To retrieve the fragment containing the 3′-UTR of *C1orf24,* the pCR2.1-TOPO construct was digested with *Xba*I. The purified fragment was subcloned into pmirGLO Dual-Luciferase miRNA Target Expression Vector (Promega Corp., Madison, WI). This vector simultaneously expresses both Renilla and Firefly Luciferase. The construct was sequenced to confirm the insertion of 3′-UTR fragment of *C1orf24* downstream of Firefly Luciferase (Luc2).

### Site-directed mutagenesis at predicted miR-106b binding site in the 3′-UTR region of the human *C1orf24*

We next introduced mutations in the seed sequence recognized by miR-106 in *C1orf24* mRNA. The mutant vector was created by replacing the seed regions (GCACTTT), cloned into pmirGLO Dual-Luciferase vector as above, to ATGTGGG by site-directed mutagenesis (QuikChange II XL kit, Agilent Technologies, La Jolla, CA). Oligonucleotides containing the mutated miR-106b binding site were designed using the PrimerX, a program that automate designs mutagenic PCR primers for site-direct mutagenesis (http://www.bioinformatics.org/primerx/). The oligonucleotides were designed as follow: sense: 5′ - TGTGCAATCTT CTTGTATGTGGGAAGGCTTTTTAATTTTG-3, antisense: 5′-CAAAATTAAAA AGCCTTCCCACATACAAGAAGATTGCACA-3′. Plasmid DNA was subsequently sequenced to confirm the presence of desired mutations and to exclude potential unwanted mutations.

### Dual-luciferase reporter assay in PCCL3 cell line

Cells were seeded onto 24-well tissue culture plates at a cell density 8 × 10^4^. Transient co-transfection with either the wild type (*wt*) or mutated (*mut*) constructs and miR-106b or negative control mimics were performed using siPORT neoFX (Ambion). Forty-eight hours after transfections, cells were washed with PBS and incubated with Passive Lysis Buffer. Firefly and Renilla luciferase activities were measured using Dual-Luciferase Reporter Assay System (Promega Corp.) using a luciferase multi-well plate reader Wallac Victor III (PerkinElmer, Waltham, MA), according to manufacturer protocol. Results are expressed as Firefly/Renilla luciferase ratios.

### Apoptosis assay

We tested whether knockdown of *C1orf24*, by transfection of cells with miR-106b, would induce apoptosis. About 6.6 × 10^4^ WRO and 10^5^ TPC1 cells were transiently transfected either with miR-106b mimic, si-*C1orf24* or respective scramble negative controls using siPORT neoFX transfection agent, according to manufacturer’s instructions (Ambion), and cell electroporation (150 V, 900 μF), respectively. After 72 hours of WRO transfection and 48 hours of TPC1, cells were double-stained with Annexin V and Nexin 7-AAD according to manufacturer’s recommendations (Millipore Corp., Billerica, MA). Cell-associated fluorescence was analyzed by Guava EasyCyte Mini flow cytometer (Millipore Corp.). Results are expressed as percentage of apoptotic positive cells. Both early apoptotic (annexin V-positive) and late apoptotic (annexin V- and 7 AAD-positive) cells were included in the analysis. Experiments were performed in quintuplicates.

### Scratch wound healing assay

Scratch assay was used to investigate the effects of miR-106b expression on cell migration. Briefly, 6.6 × 10^4^ WRO and 1.9 × 10^5^ TPC1 cells were transiently transfected with miR-106b mimic, si-*C1orf24* or respective scramble negative controls using siPORT neoFX transfection agent to WRO cells according to manufacturer’s instructions (Ambion) and cell electroporation (150 V, 900 μF) to TPC1 cells. After 24 hours of transfection, the “wound gap” was introduced, by scraping the cell monolayer with a pipette tip, into WRO and TPC1 cultures. Then, the medium was replaced and incubated in a humidified chamber at 37°C with 5% CO_2_, integrated with Zeiss Axio Observer Z1 inverted phase microscope system and equipped with a MRc camera (Carl Zeiss, Göttingen, Germany). Images at time zero were captured to record the initial area of wounds. Subsequently, images were taken every one hour, over the course of 72 h, at 5x magnification. Cell migration toward the wounds was expressed as percentage of wound closure. Area of the wound was quantified using CorelDraw Graphics Suite X5 (Ottawa, Canada). Experiments were performed in quintuplicates.

### Cell cycle analysis

WRO (4 × 10^4^) and TPC1 (10^5^) cells were seeded in 24-well dishes. After synchronization of the cells by serum starvation for 24 h, cells were replaced with DMEM medium, in WRO cell line, and RPMI medium, in TPC1 cell line, supplemented with 10% FBS for next 24 h. Cells were fixed in 70% ethanol for 1 h, labeled with Guava Cell Cycle Assay reagent and analyzed using Guava PCA flow cytometer (Guava Technologies) according to manufacturer’s recommendation. Experiments were performed in quintuplicate.

### Statistical analysis

Relative expression levels and band intensities were compared using Mann-Whitney U test. Firefly and Renilla Luciferase activities ratio was calculated by One-Way ANOVA (Bonferroni post-hoc test). Differences in apoptotic rate, wound closure and cell cycle assay were analyzed by student’s T-test and by its non-parametric analysis of Mann-Whitney U test, when data were not found to be normally distributed. All statistics were calculated using GraphPad PRISM (Version 5.0; San Diego, CA). *P* value < 0.05 was considered statistically significant.

## SUPPLEMENTARY MATERIAL FIGURES




