# Supplementary material for: microRNA-106b-mediated down-regulation of C1orf24 expression induces apoptosis and suppresses invasion of thyroid cancer
Source: Oncotarget. 2015 Jul 22;6(29):28357–70. doi: 10.18632/oncotarget.4947 (PMC4695065; doi:10.18632/oncotarget.4947)
Supplement: Supplementary file 1 [file oncotarget-06-28357-s001.pdf]

**microRNA-106b-mediated down-regulation of *C1orf24* expression induces apoptosis and suppresses invasion of thyroid cancer**

**Supplementary Material**

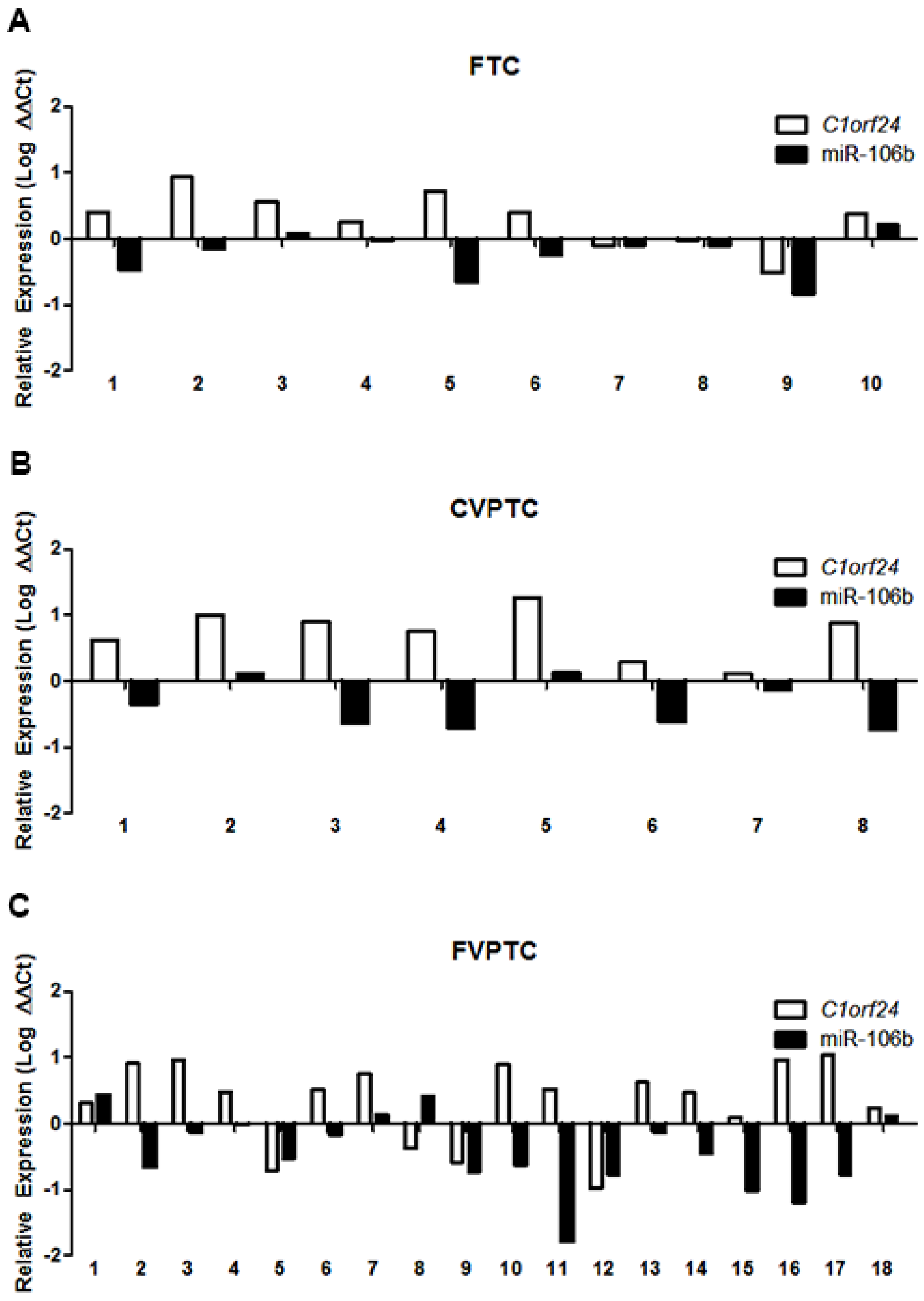


**Supplementary Figure S1: Relative Expression of *C1orf24* and miR-106b in each thyroid carcinoma sample:** (A) Follicular thyroid carcinomas (FTC, *n*=10). (B) Classical variant of papillary thyroid carcinomas (CVPTC, *n*=8). (C) Follicular variant of papillary thyroid carcinomas (FVPTC, *n*=18).

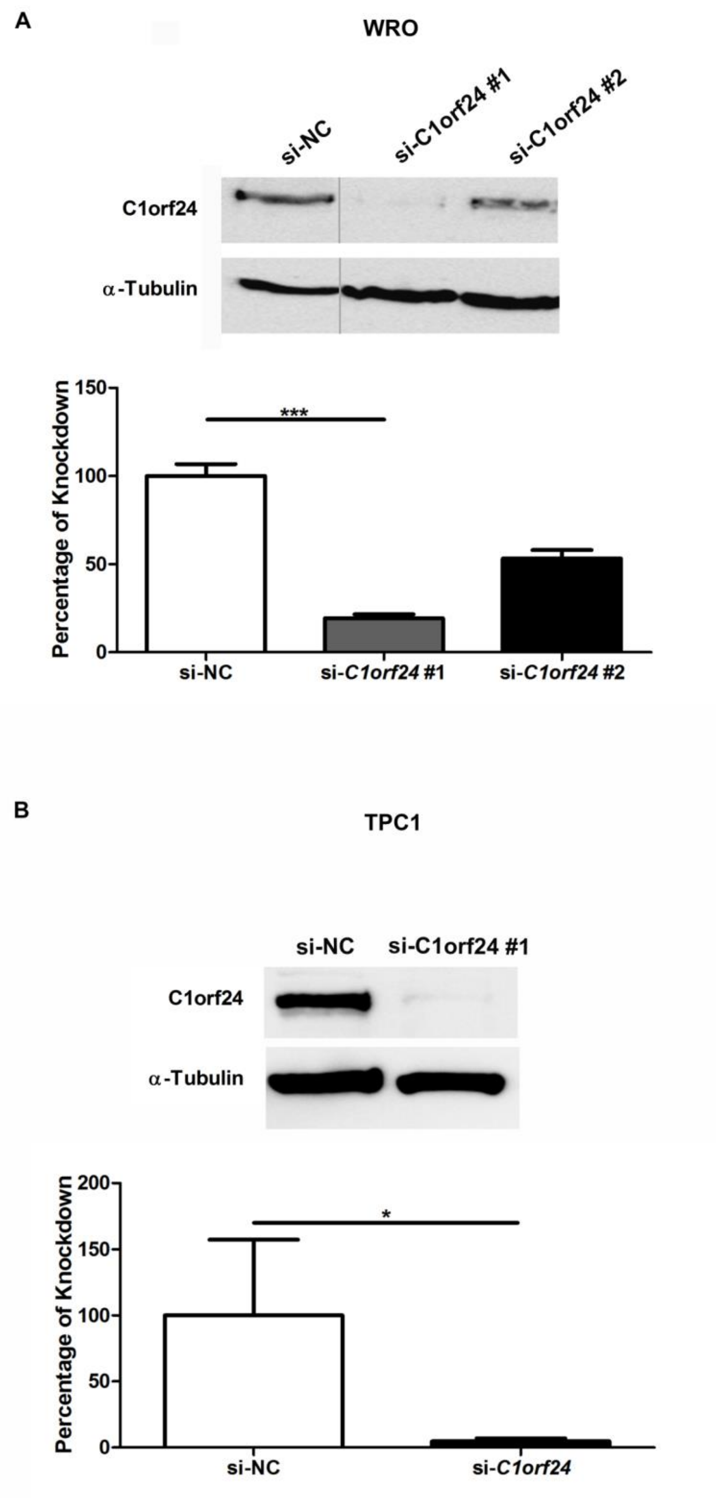


**Supplementary Figure S2: siRNA-mediated C1orf24 knockdown in thyroid carcinoma cell lines**. C1orf24 protein levels were reduced in both WRO (A) and TPC1 (B) cell lines transfected with si-*C1orf24* ($P<0.001$ and $P=0.0415$, respectively). α-tubulin was used as a loading control. The band intensities were quantified and normalized to α-tubulin intensities. Results are graphically represented (bottom of each Figure).
